# Virtual organelle self-coding for fluorescence imaging via adversarial learning

**DOI:** 10.1117/1.JBO.25.9.096009

**Published:** 2020-09-29

**Authors:** Thanh Nguyen, Vy Bui, Anh Thai, Van Lam, Christopher B. Raub, Lin-Ching Chang, George Nehmetallah

**Affiliations:** a The Catholic University of America, Electrical Engineering and Computer Science Department, Washington, DC, United States; b The Catholic University of America, Biomedical Engineering Department, Washington, DC, United States

**Keywords:** artificial intelligence, microscopy, fluorescence imaging

## Abstract

**Significance:** Our study introduces an application of deep learning to virtually generate fluorescence images to reduce the burdens of cost and time from considerable effort in sample preparation related to chemical fixation and staining.

**Aim:** The objective of our work was to determine how successfully deep learning methods perform on fluorescence prediction that depends on structural and/or a functional relationship between input labels and output labels.

**Approach:** We present a virtual-fluorescence-staining method based on deep neural networks (VirFluoNet) to transform co-registered images of cells into subcellular compartment-specific molecular fluorescence labels in the same field-of-view. An algorithm based on conditional generative adversarial networks was developed and trained on microscopy datasets from breast-cancer and bone-osteosarcoma cell lines: MDA-MB-231 and U2OS, respectively. Several established performance metrics—the mean absolute error (MAE), peak-signal-to-noise ratio (PSNR), and structural-similarity-index (SSIM)—as well as a novel performance metric, the tolerance level, were measured and compared for the same algorithm and input data.

**Results:** For the MDA-MB-231 cells, F-actin signal performed the fluorescent antibody staining of vinculin prediction better than phase-contrast as an input. For the U2OS cells, satisfactory metrics of performance were archieved in comparison with ground truth. MAE is <0.005, 0.017, 0.012; PSNR is >40/34/33  dB; and SSIM is >0.925/0.926/0.925 for 4′,6-diamidino-2-phenylindole/hoechst, endoplasmic reticulum, and mitochondria prediction, respectively, from channels of nucleoli and cytoplasmic RNA, Golgi plasma membrane, and F-actin.

**Conclusions:** These findings contribute to the understanding of the utility and limitations of deep learning image-regression to predict fluorescence microscopy datasets of biological cells. We infer that predicted image labels must have either a structural and/or a functional relationship to input labels. Furthermore, the approach introduced here holds promise for modeling the internal spatial relationships between organelles and biomolecules within living cells, leading to detection and quantification of alterations from a standard training dataset.

## 1 Introduction

Microscopy techniques, particularly the family of epifluorescence modalities, are workhorses of modern cell and molecular biology that enable microscale spatial insight. Intensity- and/or phase-based microscopy techniques such as brightfield, phase contrast, differential interference contrast, digital holography, Fourier ptychography, and optical diffraction tomography,^1^^–^^7^ among other modalities, have the potential to visualize the subcellular structure. However, these methods depend largely on light scattering that is defined by the internal structure-based index of refraction, which lacks biomolecular specificity. Fluorescence-based techniques, on the other hand, excite fluorophores, which act as labels to spatially localize biological molecules and structures within cells. These imaging techniques, especially fluorescence approaches, involve time-consuming preparation steps and costly reagents, introduce the possibility of signal bias due to photobleaching, and in time-lapse vital imaging, allow for possible misinterpretation of cell behavior due to gradual accumulation of sublethal damage from intense ultraviolet and other wavelengths used to excite molecular labels.^8^ Thus microscopy of cells is challenging due to the inherent trade-offs in sample preservation, image quality, and data acquisition time and the variability between labeling experiments.

Deep convolutional neural networks (DCNNs) capture nonlinear relationships between images globally and locally, resulting in significantly improved performance for image processing tasks compared with the traditional machine learning methods. In many studies of cells requiring subcellular details, fluorescence labels specific to selected biomolecules or organelles are imaged simultaneously or in rapid sequence in separate fluorescent signal channels to assign subcellular localization of biomolecules to certain organelles. Alternatively, co-registered phase contrast or other brightfield modality images are acquired to relate biomolecular localization to cell morphology and structural features. In several recent studies, applications of DCNNs to fluorescence microscopy of cells investigated the performance of these algorithms in super-resolution,^9^^–^^12^ image restoration,^13^ image analysis,^14^ and virtual histological staining.^15^ The major goal of these studies was to reveal additional image information content based on the statistical model of the specific DCNN linking the training dataset to ground truth images to be predicted from test data. Recently, DCNNs have been employed to create digital staining images by training a pair of images to transform transmitted light microscopic images into fluorescence images^16^^,^^17^ and quantitative phase images into equivalent bright-field microscopy images that are histologically stained.^18^

Based on these results, we formulated two questions leading to null and alternative hypotheses. First, does model prediction performance depend on the image modality and subcellular labels selected for training and prediction? Second, do errors in predicted images contribute to the likelihood of misinterpreting biology based on the image predictions? To address these questions, we developed a DCNN-based computational microscopy technique employing a customized conditional generative adversarial network (cGAN) that models the relationships between optical signals acquired using any imaging modality or fluorescence channel, assuming the signals are co-registered. This technique was employed to fulfill two objectives addressing the hypotheses. First, we determined image prediction performance using several fluorescence channels and phase contrast images for training and compared prediction performance between sets of input and output optical signals and fluorescence labels for two independent datasets using different cell lines. Second, we compared predicted images with ground truth to identify signal error, evaluating the error fractions important and unimportant to biological interpretation through a novel quantitative prediction performance parameter. To further demonstrate the adaptability of the cGAN algorithm to different image prediction tasks, out-of-focus fluorescence images of cells were digitally refocused after application of a trained, end-to-end ranged autofocusing (AF) algorithm.

The results of this work support prediction of fluorescent labeling of cells from other image data. These predictions have the potential, in one application, to significantly reduce the cost and the effort in preparation of cell imaging experiments to end-users by replacing or supplementing actual labeling experiments. This first application requires a high degree of fidelity of predicted images to ground truth, within a tolerance of error that allows for accurate biological conclusions to be drawn. Here we introduce a tolerance level to quantify the fidelity of predicted images. A second application lies in removing artifacts and aberrations from existing image datasets. Here we demonstrate an AF method that improves blurred regions by reference-free metrics. Other applications are discussed, including transfer learning to ascertain the effects of a treatment on cells by direct comparison of actual fluorescence labels versus predictions from controls. Thus the described new method supports cell studies based on mixed virtual cell imaging. Because the VirFluoNet method is digital, it significantly reduces the possibility for cell damage from phototoxicity and signal degradation from photobleaching. This work also extended cGAN capabilities to the prediction of fluorescent images. We envision that the approaches of this study will be useful for prescreening large fluorescence microscopy datasets, identifying and correcting out-of-focus regions, and potentially saving time in multilabel fluorescence experiments by predicting some fluorescence label features from other labels signals. Due to the rapid development of hardware/platform (in terms of higher speed, lower cost, and smaller size) supporting machine learning applications, the algorithms of this study should become useful in an expanding number of applications. A future application of the approach may be the study of complex protein structures and modeling protein–protein or protein–organelle relationships.^16^^,^^17^

## 2 Method

The order of methods and results, below, follows the order of the first and second objectives described above. However, the ancillary goal of demonstrating refocusing of out-of-focus image data using a cGAN model is presented first, as this was an image preprocessing step contributing to high-quality training data for fluorescent image transforms, which would be described later.

### 2.1 Summary of Approach

To achieve AF as a preprocessing step for training data, we trained and tested the customized cGANs on a dataset of U2OS cells with labeled F-actin (U2OS-AF) and used the refocused images to enhance the input images before training models of Fluo-Fluo 2, 3, 4, defined below.

**Fig. 1:**
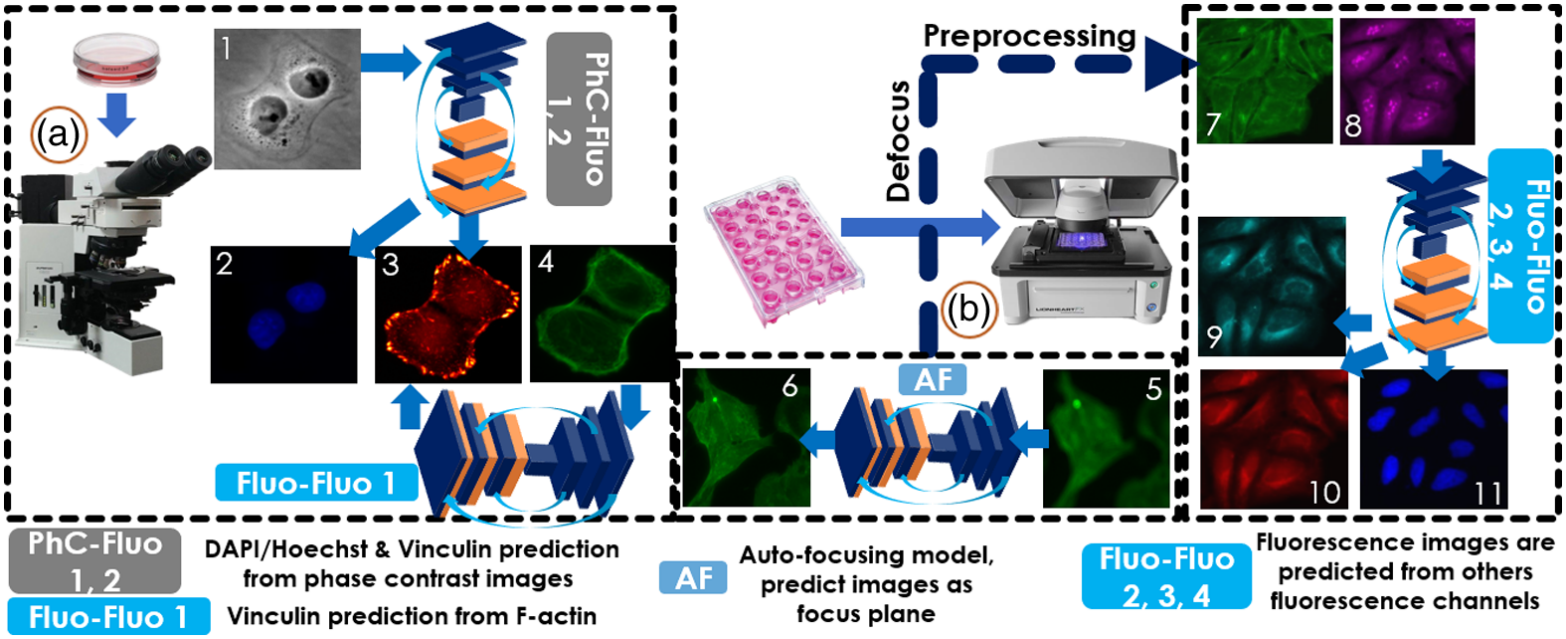
Virtual fluorescence imaging pipeline for cell microscopy with all models implemented in our study. MDA-MB-231 cells (on the left) were imaged to collect co-registered phase contrast, DAPI, F-actin, and vinculin. The PhC-Fluo models were trained to predict fluorescence images from phase contrast (either DAPI or vinculin). The Fluo-Fluo models using MDA-MB-231 in Fluo-Fluo model 1 and U2OS-CPS in Fluo-Fluo models 2, 3, 4 (on the right) predicted florescence images from a co-registered fluorescence channel, from the same cells. Although each model performs different goals in our study, out-of-focus images were fed to the AF model (at center) as a preprocessing step to refocus the cell images (U2OS-AF) before reuse in training and testing of the Fluo-Fluo models. (1) Phase contrast, (2) DAPI, (3) vinculin, (4) F-actin, (5) out-of-focus F-actin, (6) in-focus predicted Factin, (7) Golgi apparatus plasma membrane F-actin, (8) nucleoli and cytoplasmic RNA, (9) endoplasmic reticulum, (10) mitochondria, and (11) DAPI/Hoechst. (a) Regular fluorescence imaging for MDA-MB-231 cells collection and (b) automated cellular imaging system for U2OS-CPS/AF data collection. Details of sample preparation are described in Sec. 2.2.

For the first objective, we trained two cGAN models (PhC-Fluo 1, 2) to generate 4′,6-diamidino-2-phenylindole (DAPI) and vinculin from phase contrast images, respectively, and four cGAN models (Fluo-Fluo 1, 2, 3, 4) for fluorescence prediction from another fluorescence image (see Fig. 1). The training and testing data for these models were co-registered with phase contrast and fluorescence images of human breast cancer cells from the MDA-MB-231 cell line and multiple fluorescence channels corresponding to specific organelle labels of human osteosarcoma cells from the U2OS cell line labeled with a standard Cell Painting protocol (U2OS-CPS).

For the second objective, training datasets of the PhC-Fluo 2 and Fluo-Fluo 1 models were evaluated for absolute image error (ground truth minus predicted image, always positive). The ground truth and prediction channels were vinculin immunostaining, paired with either co-registered phase contrast or F-actin fluorescence label channels. Not all parts of the absolute image error contribute equally to image misinterpretation. The absolute image error was split into two components, spatial/area error and pixelwise intensity error, by summing segmented pixel area and intensity differences from a thresholded absolute error map. The threshold was scanned through the full map bit depth. A global minimum total weighted error was determined from the weighted sum of the two individual error terms.

### 2.2 Data Preparation

#### 2.2.1 MDA-MB-231 breast cancer cell

The human breast cancer cell line MDA-MB-231 provided by Dr. Zaver Bhujwalla (Johns Hopkins School of Medicine, Baltimore, MD) was cultured on tissue culture-treated polystyrene dishes, in standard tissue culture conditions of 37°C with 5% ${\mathrm{CO}}_{2}$ and 100% humidity (HERAcell 150i, Thermo Fisher Scientific, Waltham, MA). Cells were fed with Dulbecco’s Modified Eagle Medium supplemented with 10% Fetalgrow (Rocky Mountain Biologicals, Missoula, Montana) and 1% penicillin–streptomycin (Corning Inc., Corning, New York, NY). Cells were fed every two days and passaged using trypsin (Mediatach, Inc. Manassas, VA) once they reached confluence. MDA-MB-231 WT after passaging were seeded on 35-mm tissue culture treated dishes (CELLTREAT Scientific Products, Pepperell, MA), following the culture procedure provided above. After 24 h of culture, cells were washed with 1× phosphate buffered saline (PBS) (Sigma-Aldrich, St. Louis, MO) to remove cell debris and fixed with 3.7% formaldehyde diluted from 16% Paraformaldehyde (Electron Microscopy Sciences, Hatfield, PA) for 15 min, permeabilized with 0.1%Triton-X in PBS for 5 min, and blocked by horse serum for 1 h. Vinculin monoclonal (VLN01) antibody (Thermo Fisher Scientific, Rockford, IL) and integrin beta-1 (P5D2) antibody (Iowa University Department of Biology, Iowa, IA) were diluted in 1× PBS containing 1% bovine serum albumin to reach 2  μg/ml concentration. After 1 h, cells were washed and incubated for a further 1 h with antimouse secondary antibody conjugated to a fluorophore. Then cells were co-stained with a solution containing 1:1000 dilution of a 2-μg/ml DAPI (Life Technologies, Carlsbad, CA) and 1:1000 dilution of AlexaFluor-labeled phalloidin (Life Technologies Corporation, Eugene, OR) for one more hour before being washed with 1× PBS again. Cells were stored in 1× PBS at 4°C until the imaging session. Fluorescence images were acquired using an Olympus BX60 microscope (Olympus, Tokyo, Japan), with 60×, NA 1.25 oil immersion Plan Apo objective, and a Photometrics CoolSNAP HQ2 high-resolution camera (1392×1040  pixels, 6.45×6.45  μm pixels) with Meta-Morph software. The MDA-MB-231 dataset (before augmentation) contained 74 images (1392×1040  pixels, 8-bit) for training + validation and 6 images (1392×1040  pixels) for testing.

#### 2.2.2 Human osteosarcoma U2OS cell, autofocusing (U2OS-AF)

High-content screening datasets of U2OS cells used in this study were previously made publicly available.^19^ The data were acquired from 384-well microplates on an ImageXpress Microautomated cellular imaging system with Hoechst 33342 dye and Alexa Fluor 594-labeled phalloidin at 20× magnification, 2× binning and 2 sites per well. Thirty-two image sets were provided corresponding to 32 z-stacks with 2  μm between slices. Each image is 696×520  pixels in a 16-bit TIF format, LZW compression. For each site, the optimal focus was found using laser autofocusing to find the well bottom. The automated microscope was then programmed to collect a z-stack of 32 image sets covering from −32  μm to 30  μm of out-of-focus range. In total, 1536 images in 9 folders of human osteosarcoma U2OS cells with augmentation were used in this study.

#### 2.2.3 Human osteosarcoma U2OS cell, cell painting staining protocol (U2OS-CPS)

U2OS cell (#HTB-96, ATCC) raw images used here can be found in Ref. 20. Cells were cultured at 200 cells per well in a 384-wellplate. Eight different cell organelles were labeled by different stains: nucleus (Hoechst 33342), endoplasmic reticulum (concanavalin A/AlexaFluor488 conjugate), nucleoli and cytoplasmic RNA (SYTO14 green fluorescent nucleic acid stain), Golgi apparatus and plasma membrane (wheat germ agglutinin/AlexaFluor594 conjugate), F-actin (phalloidin/AlexaFluor594 conjugate), and mitochondria (MitoTracker Deep Red). Five fluorescent channels were imaged at 20× magnification using an epifluorescence microscope with illumination and excitation central wavelengths as follows: DAPI (387/447  nm), GFP (472/520  nm), Cy3 (531/593  nm), Texas Red (562/642  nm), and Cy5 (628/692  nm). The dataset contains 3456×9 (folders) of each fluorescent channel (1024×1374  pixels). Eight folders across all channels were used for training and validation. The last (ninth) folders were used for testing purposes. In total, 3456 images in 9 folders and 5 channels each of human osteosarcoma U2OS cells with augmentation were used in this study.

### 2.3 Training and Testing Data Preparation

Patch images (256×256 or 128×128) were randomly cropped from full fields-of-view to form input–output pairs for training. During training with the MDA-MB-231 breast cancer cell dataset, images were augmented with rotation and flipping to generate more features. Histogram equalization techniques were applied to enhance the image contrast (only for the MDA-MB-231 dataset). All images in the datasets were preprocessed with [−1,1] normalization only. Overlapping regions of input on phase contrast or fluorescence channels were cropped randomly in horizontal and vertical directions in the training process. The predicted/tested images were divided into subregions with some overlap between adjacent regions to be stitched into a larger FOV based on an alpha blending algorithm. Finally, the stitched predicted images were inversely normalized to the original image range. Model training required 12 h. The best models were saved based on performance using validation data (20% of training data).

### 2.4 Conditional Generative Adversarial Network Implementation

**Fig. 2:**
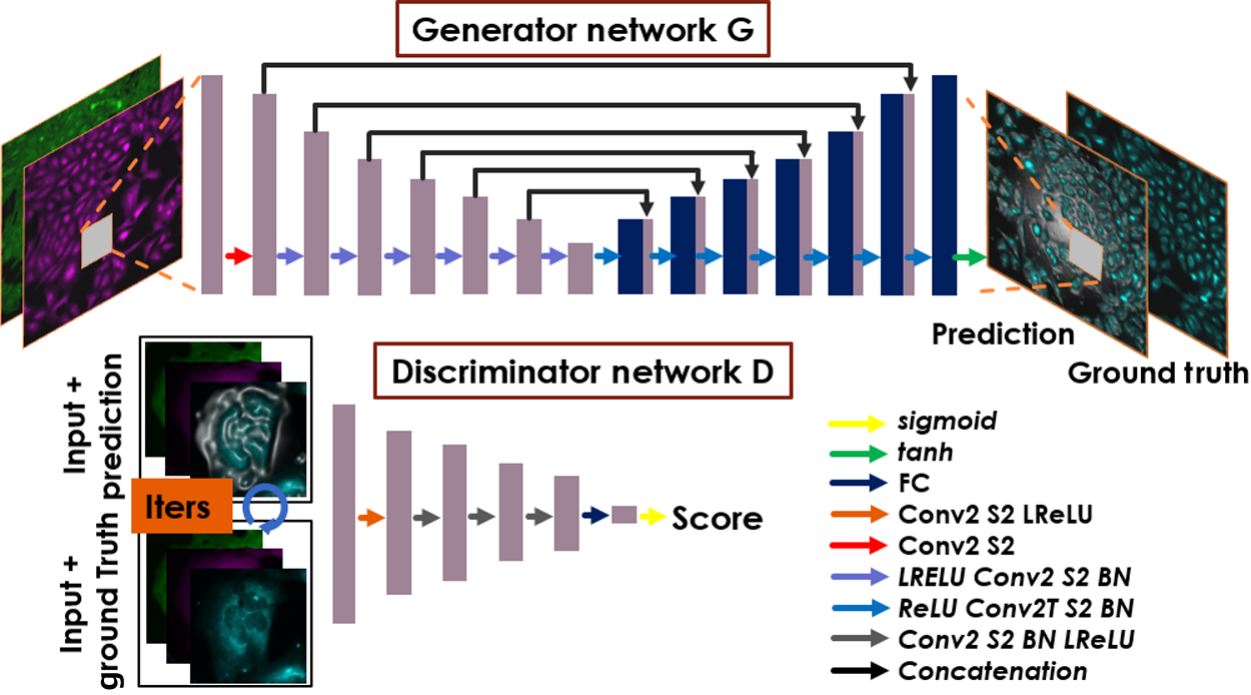
cGAN for fluorescence image prediction. This figure only shows one of the models. Golgi + F-actin fluorescence channels are the inputs to predict endoplasmic reticulum as the fluorescence images targeted for prediction. Generator network G contains an initial convolution layer with stride of 2 (S2), encoded-blocks (LReLU-Conv2S2-BN), and decoded-blocks (ReLU-Conv2TS2-BN), ending with Tanh activation and using skip connections. A generator network transforms the input images and results in predicting fluorescence images. A discriminator network D is initialized with a convolution layer stride 2, following a BN, 3 convolutional blocks (Conv2S2-BN-LReLU), one fully connected layer, and sigmoid activation. The discriminator D outputs a score of how likely the input of a group of images is good or bad. The input of discriminator D was formed as a conditional input by concatenating the predicted image or ground truth with generator $G^{\prime }s$ input.

The proposed DCNN-based cGAN takes one or a set of intensity images I as the network input and outputs a single fluorescence image representing a single targeted protein or subcellular compartment. The intensity images I are captured under phase contrast or fluorescence microscopy. The cGAN consists of two subnetworks (see Fig. 2), the generator G and the discriminator D. The generator G is trained to predict the proteins $\Phi_{G}=G(I)$ from the given input I. During the training process, the generator $G^{\prime }s$ parameters ($\theta_{G}$—weights and biases of the generator) were optimized to minimize a loss function l through N input–output training pairs:

θ^G=arg minθG∑n=1N1Nl[GθG(In,Φn)]. (1)

The generator G is a customized model based on the original U-Net model,^21^ which can adapt to efficient learning based on pixel-to-pixel transformation. A series of operations are performed, including batch-normalization (BN), nonlinear activation using ReLU/LeakyReLU (LReLU) functions, convolution (Conv2), and convolution transpose (Conv2T) layers with filters with a kernel size of k=3. This model contains an initial convolution layer with a stride of 2 (S2), encoded-blocks (LReLU-Conv2S2-BN) and decoded-blocks (ReLU-Conv2TS2-BN), and the Tanh activation function at the end.

The discriminator network D (contains weights and biases $\theta_{D}$) aims to distinguish the quality of prediction of the generator G. Discriminator D is initialized with a convolution layer stride 2, following a BN, 3 convolutional blocks (Conv2S2-BN-LReLU), one fully connected layer and sigmoid activation; filters with a kernel size of k=5 are used in the discriminator D. More details about the adversarial networks can be found in Refs. 22 and 23. The following adversarial min–max problem in terms of expectation was solved to enhance the generator $G^{\prime }s$ performance:

$$\min_{\theta_{G}}\;\max_{\theta_{D}}E_{I,\Phi \;}[\log\;D_{\theta_{D}}(I,\Phi )]+E_{I}(\log\{1-D_{\theta_{D}}[I,G(I)]\}).$$ (2)

The motivation of using discriminator D is to preserve the high-frequency content of the predicted images. Using the conventional loss functions such as the mean absolute error (MAE), peak-signal-to-noise ratio (PSNR), and structural similarity index (SSIM), the minimization of these pixel-wise loss functions will lead to solutions that have less perceptual quality. By training the generator G along with the discriminator D, the generator G can learn to generate realistic images of protein prediction in case the input–output image pairs are not strongly correlated. For that purpose, the proposed perceptual loss function l is defined as a weighted sum of separate loss functions:

$$l=\lambda_{1}l_{\mathrm{MAE}}+\lambda_{2}l_{G}+\lambda_{3}l_{\theta_{G}},$$ (3)

where

$$l_{\mathrm{MAE}}=\frac{1}{W\times H}\left\||\Phi |-|G_{\theta_{G}}(I)|\right\|,$$ (4)

$$l_{G}=-\log\;D_{\theta_{D}}[I,G(I)],$$ (5)

$$l_{\theta_{G}}=\|\theta_{G}\|,$$ (6)

where ‖.‖ denotes the $L_{1}$-norm; $\left(\lambda_{1},\lambda_{2},\lambda_{3}\right)$ are the hyper parameters that control the relative weights of each loss components and were choose as ($\lambda_{1}=0.99$, $\lambda_{2}=0.01$, and $\lambda_{3}=0.001$; and W×H is the input image size. The adaptive momentum optimizer was used to optimize the loss function with a learning rate of $2\times 10^{-4}$ for both the generator and the discriminator models. These models were implemented using a Tensorflow framework on GPU RTX 2070 16 GB RAM Intel Core i7 and GPU Titan Xp 8 GB RAM Intel Core i7, and the models were selected based on the best performance on the validation dataset.

### 2.5 Quantitative Index of Predicted Image Error

For evaluating vinculin signal prediction using PhC-Fluo 2 and Fluo-Fluo 1, a quantitative index of predicted image error versus ground truth was defined as the weighted sum of normalized pixel-wise intensity and spatial errors, computed over a range of tolerances of the 8-bit absolute difference error (8-bit range in the MDA-MB-231 dataset). The rationale for this index was that epifluorescence images of cell labels are qualitative in pixel intensity, to a certain tolerance, due to photobleaching and differences in experimental preparation, microscope instrument parameters, and camera settings. Therefore, small differences between pixel intensity values of predicted images and ground truth (intensity errors) are less likely to produce misinterpretations than a predicted signal where there is no true signal or the lack of a predicted signal where there is a true signal (spatial errors). Segmentation of absolute difference error maps so that error pixels above a certain tolerance are bright green highlights both errors. The sum of these two error terms, weighting each equally here for illustrative purposes, is minimized at a single error tolerance level:

$$\mathrm{TL}=\min[\beta_{1}\times IE(O,G,i)+\beta_{2}\times SE(O,G,i)],$$ (7)

where TL is the tolerance level; O, G are the input/predicted image and ground truth, respectively; i is the bit-depth threshold percentage crossing from 0% to 99% of the bit-depth range of the image; $\beta_{1}$, $\beta_{2}$ are weights (set as equal in this study); and IE is the intensity error function that measures the MAE of two images below the threshold level and is normalized by the maximum bit depth. SE is the binary segmented error function that measures the area fraction of error outside of tolerance at a given threshold level. This second error function quantifies the area of ground truth signal that is much brighter than in the predicted image at the same pixel, and vice versa.

## 3 Results

In this section, we describe the results from all implementations in three categories: (1) AF: refocusing out-of-focus images; fluorescence prediction from (2) phase contrast images and (3) fluorescence images, and (4) error quantification. We combined (2) and (3) into one section for comparative purposes. Although (1) was the preprocessing objective, (2) + (3) address the first objective, and (4) addresses the second objective.

### 3.1 Autofocusing

**Fig. 3:**
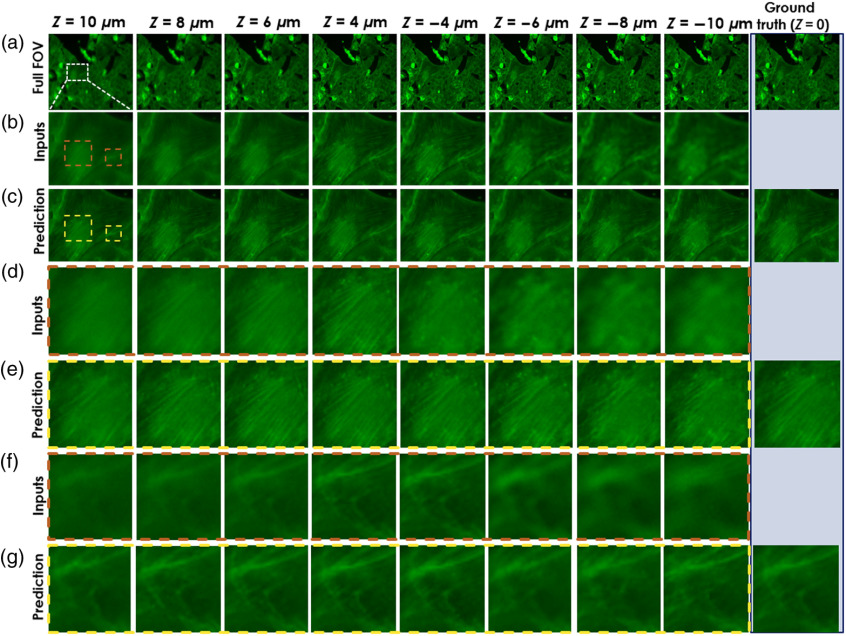
Deep-learning-based fluorescence channel AF. AF model is tested on U2OS-AF dataset. (a) The full FOV of a test image at different z depths, (b) the zoom-in areas as the inputs, (c) the zoom-in area prediction, and (d)–(g) the different zoom-in areas (marked by colored boundaries) of input and prediction, respectively. The ground truth column is put on the right with corresponding views of input and prediction for comparison.

We trained a DCNN model, named “AF model”, to predict focused images from blurred out-of-focus images. The model was trained and tested on the F-actin channel of a U2OS-AF dataset before applying the DCNN on out-of-focus images used in the Fluo-Fluo 2, 3, 4 model’s training and testing (see Fig. 1). The model took a single out-of-focus and a focused image as a pair of input–output samples for training. Images in the U2OS-AF dataset were acquired from one 384-well microplate containing U2OS cells stained with phalloidin at 20× magnification, 2× binning, and 2 sites per well. To support the Fluo-Fluo models with the U2OS-CPs dataset, we chose only a fixed out-of-focus range [−10  μm,10  μm] in the U2OS-AF dataset that covers the whole range of out-of-focus levels in the U2OS-CPS dataset. We do not use images located inside the [−2  μm,2  μm] range from the focus plane since they appeared nearly as focused as the ground truth (z=0). In fact, it was hard to distinguish where the focus planes are for the data in the range [−2  μm,2  μm]. Notice that the focused image was repeated in many input–output pairs for different out-of-focus images at different axial planes. Figure 3 shows predicted results on testing data of U2OS-AF with different out-of-focus distances with several zoom-in regions of the most out-of-focus distances. U2OS datasets were described in Sec. 2.2.

**Fig. 4:**
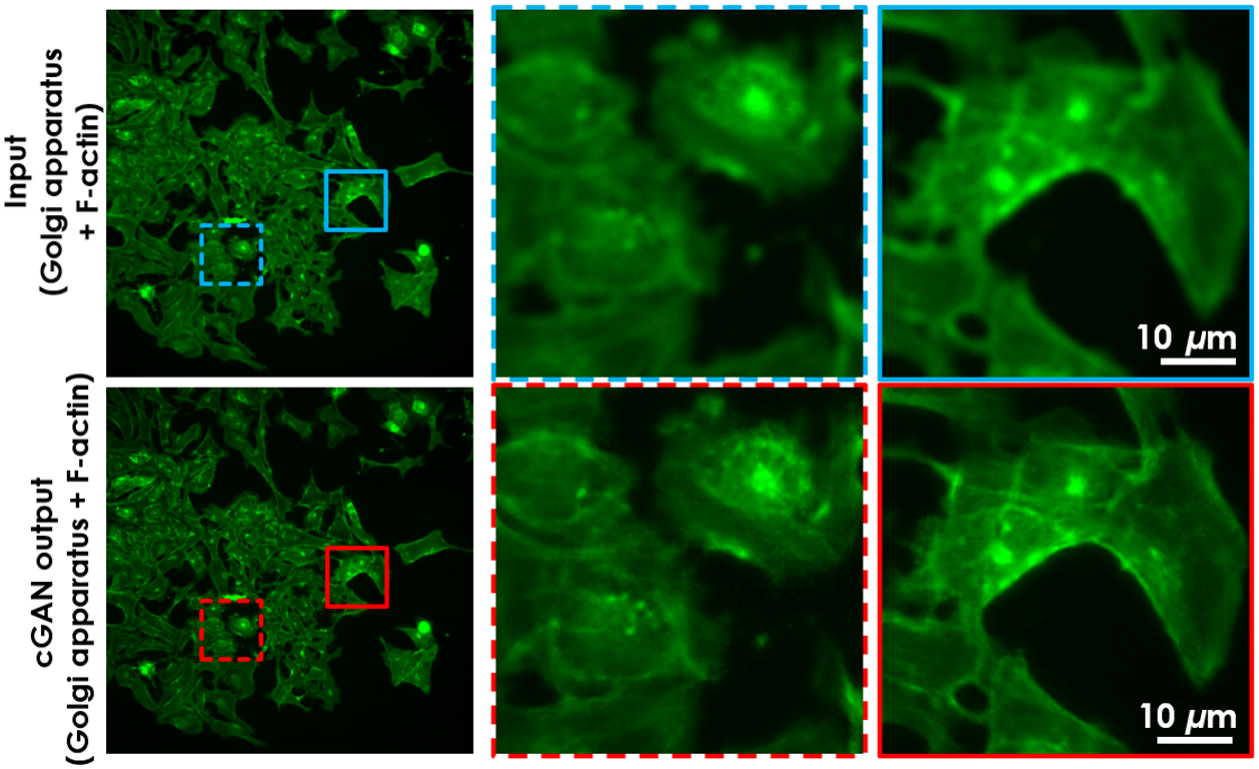
Deep-learning-based AF fluorescent prediction on unseen dataset. AF model was trained on the U2OS-AF dataset and predicted directly on out-of-focus (blur) Golgi apparatus + F-actin in the U2OS-CPS dataset, which is not seen by the AF model. Result image shows much sharper features compared with input. This is the preprocessing step that we performed on the Golgi apparatus + F-actin channel of all blur images on U2OS-CPS before training the Fluo-Fluo 2, 3, 4 models.

The AF model’s performance was evaluated by the MAE, PSNR, and SSIM^24^ on 64 predicted image groups (contains 8 different depth images) with corresponding ground truth fluorescence images (at focus plane) of each group in U2OS-AF (Fig. S1 in the Supplementary Material). The lower MAE, higher PSNR, and closer to one SSIM values are expected to relate to better performance. In order, average scores were **0.01**/0.012, **37.56**/35.639, and **0.924**/0.9 for MAE, PSNR, and SSIM, respectively. Scores in bold, which indicates “good” performance, demonstrate the feature enhancement from the AF model, compared with the scores on the right using input data that was not autofocused. The U2OS-AF model generated images that resemble the ground truth more closely than the input. Next, the AF model was used to perform the prediction on out-of-focus subset data chosen manually from the U2OS-CPS dataset to preserve the number of data samples. There is no ground truth for the focused images in the U2OS-CPS dataset, but we qualitatively evaluated the success of the network from observations of the level of focus versus depth frames and then used these images for training and analysis. Typical results are shown in Fig. 4. Moreover, for comparison purposes, we used two well-known blind (since we do not know the ground truth in the unseen dataset) spatial quality evaluators to evaluate the performance of our DL-based defocusing technique, namely, the Blind/Referenceless Image Spatial Quality Evaluator (BRISQUE) that uses a support vector regression^25^^,^^26^ and the Blind Image Quality Evaluation (PIQE) that uses perception-based features.^27^^,^^28^ Both of these techniques were applied to some of the raw and predicted images by the DL algorithm and showed ∼40% and ∼65% improvement in PIQE and BRISQUE scores, respectively, validating the qualitative evaluation.

### 3.2 Phase Contrast/Fluorescence to Fluorescence

**Fig. 5:**
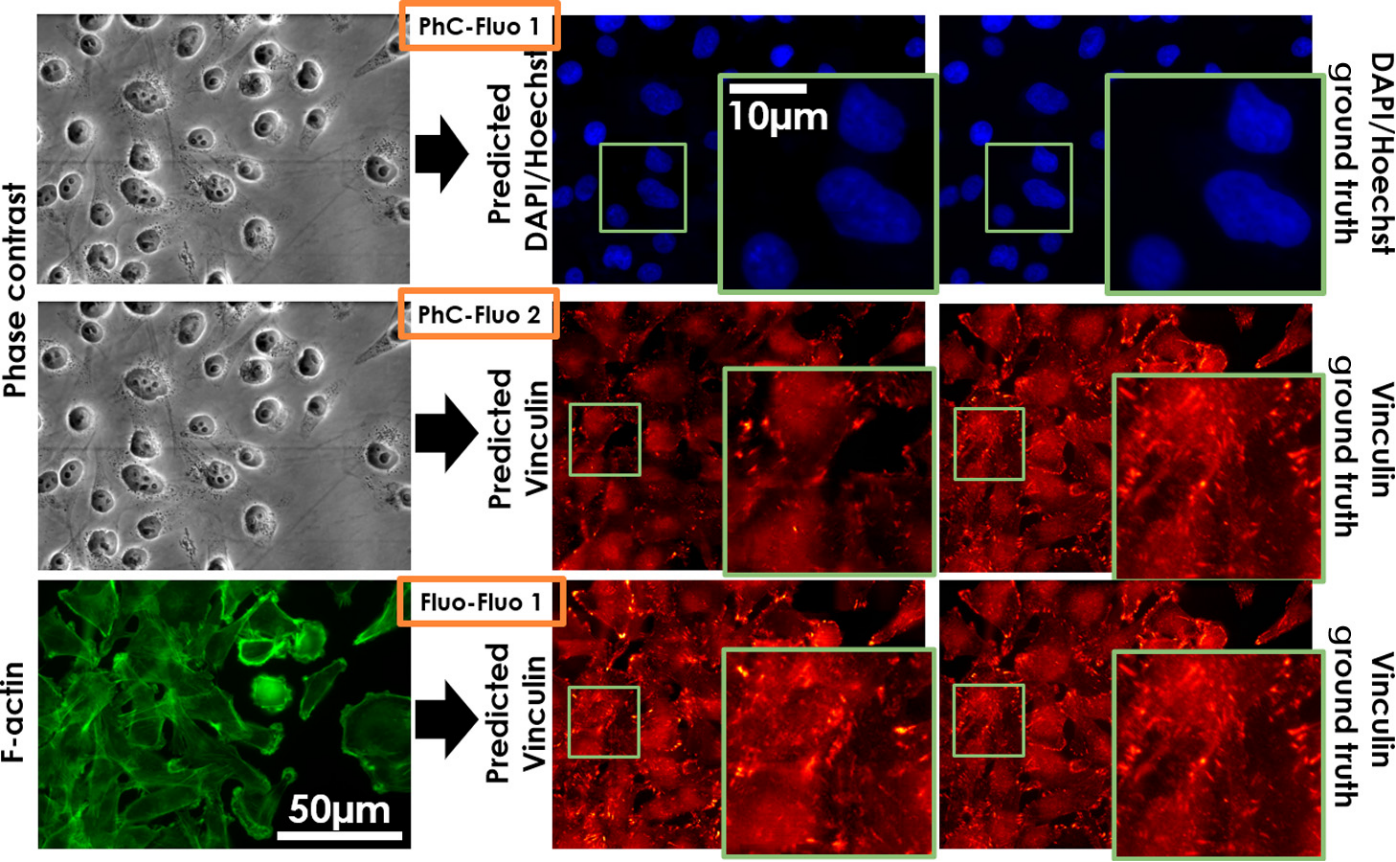
Deep-learning-based fluorescence signal prediction from the MDA-MB-231 dataset on testing data. The first two rows are representative predictions of DAPI/Hoechst (PhC-Fluo 1), vinculin (PhC-Fluo 2), respectively, from phase contrast image inputs. The third row is prediction of vinculin from F-actin (Fluo-Fluo 1).

**Fig. 6:**
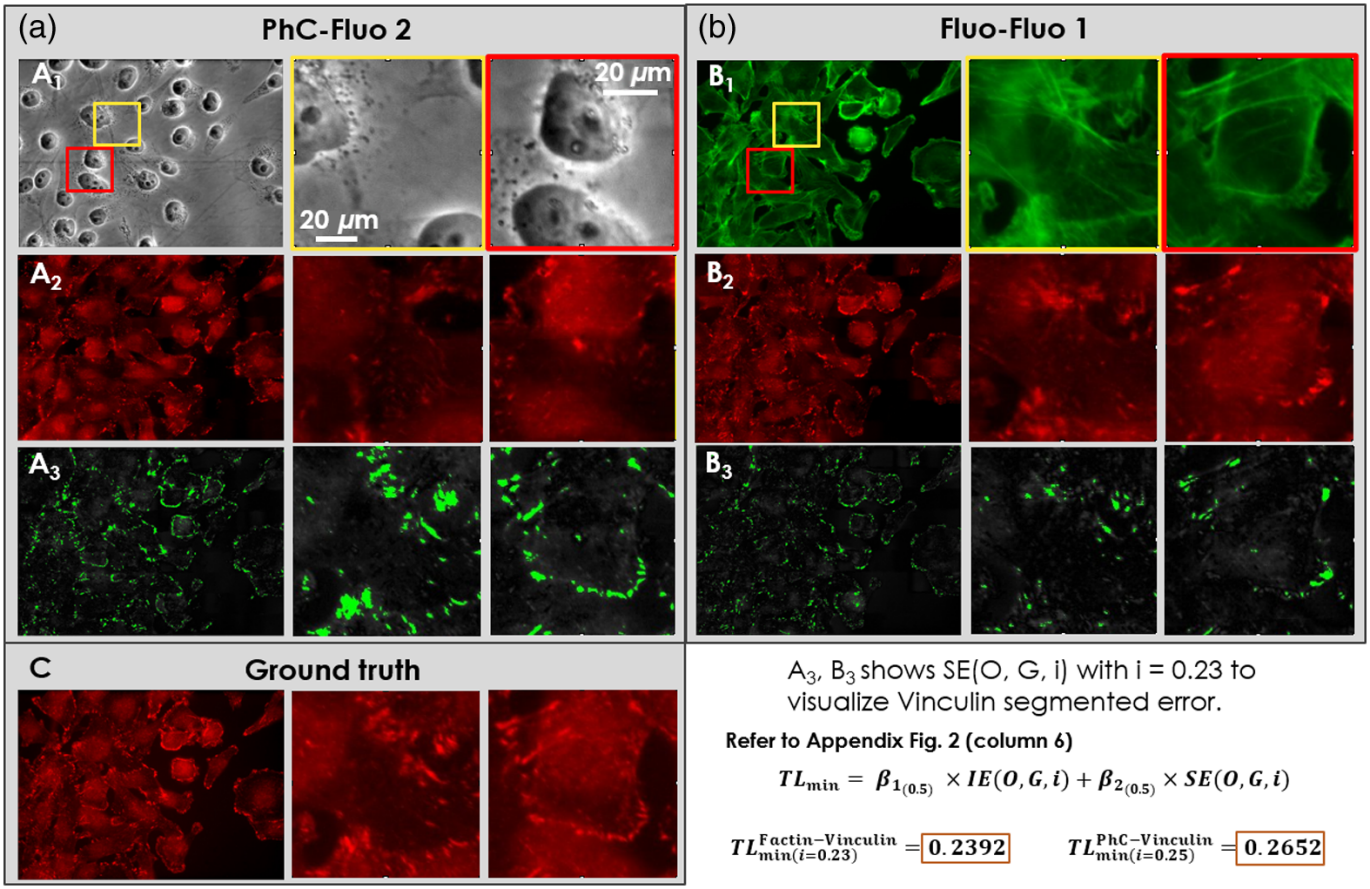
Comparison of representative input and predicted images between PhC-Fluo 2 and Fluo-Fluo 1 to predict vinculin label based on (a) phase contrast and (b) F-actin label inputs. The full field-of-view (leftmost columns) and zoom-in regions (middle and rightmost columns) of (a1) phase contrast image as the model’s input, (a2) vinculin prediction using the PhC-Fluo 2 model, and (a3) segmented error (green signal) between ground truth and predicted vinculin, used an error intensity-based threshold set at 23% of 255 bit depth; and ($b_{1}$) F-actin images as the model’s input, ($b_{2}$) vinculin prediction using the Fluo-Fluo 1 model, and ($b_{3}$) segmented error (green signal) between ground truth and predicted vinculin, using the same error intensity threshold of 23% of 255 bit depth. (c) The corresponding ground truth of vinculin signal. The minimum tolerance level measured from these images can be read directly from the graph in Fig. S2 in the Supplementary Material, column 6, with calculation described in Sec. 2.5.

Following the training phase, the trained PhC-Fluo and Fluo-Fluo networks were evaluated in a blinded fashion using testing data separated from training data. Figure 5 shows results from the breast cancer MDA-MB-231 cell line corresponding to the PhC-Fluo 1, 2 and Fluo-Fluo 1 models. With the same amount of data and training, predicted results were similar to the ground truth in the case of the DAPI/Hoechst signal predicted from PhC (PhC-Fluo 1, first row in Fig. 5). Predicted vinculin label signals from phase contrast images were not similar to the ground truth (PhC-Fluo 2, second row in Fig. 5). Meanwhile, vinculin’s punctate pattern and location at the end of actin stress fibers were determined with more accuracy from F-actin (Fluo-Fluo 1, third row in Fig. 5) than phase contrast. The predicted fluorescence results of these four deep learning models presented in Fig. 5 show nonrobustness in using the cGAN-based framework to predict complex fluorescence structures such as F-actin and vinculin from phase contrast. However, better performance was achieved in predicting vinculin from F-actin. Figure 6 shows vinculin predictions from the PhC-Fluo 2 and Fluo-Fluo 1 models, with absolute pixel-wise error between ground truth and predicted vinculin determined as the sum of area error and intensity error. The green area is a binary mask of the absolute error map after thresholding at a bit level of 50 (23% of 255 bit depth), indicating that F-actin helps predict the location of vinculin signal slightly better than phase contrast images as training inputs. Interestingly, the sum of equally weighted area and intensity errors produces a global minimum error at a certain threshold level/tolerance. To evaluate vinculin signal prediction, we used a customized matrix of performance (see Sec. 2.5). Predicted vinculin fluorescence images were closer to ground truth, assessed by the minimum tolerance level of Eq. (7), using F-actin fluorescence images as inputs (TL=0.25±0.3) than using phase contrast images as inputs (TL=0.27±0.4) (Student’s paired t-test, p<0.01).

Similarly, the Fluo-Fluo 2, 3, 4 models were implemented with the same cGAN framework. The difference between these models and Fluo-Fluo 1 is in the use of inputs that contain two fluorescently labeled subcellular compartments to predict the targeted protein. We trained and tested these models on the human U2OS-CPS cell dataset. These models can be used or edited as pretrained models with or without transfer learning on completely new types of data, thus making the proposed technique generalizable. Transforming one fluorescence channel into another can be based on one channel input-to-one channel output pairs. However, the success of model training depends on the correlation of the selected pairs, i.e., strongly correlated pairs of input–output data allow the model to learn a pixel-to-pixel transformation that is governed by the regularization of the network. This data-driven cross-modality transformation framework is effective because the input and output distributions share a high degree of mutual pixel-level information content, with an output probability distribution that is conditional upon the input data distribution.^10^ Experiments to compare the model performance based on the choices of different input–output pairs or combined inputs of phase contrast and fluorescence images would be an interesting future research topic.

**Fig. 7:**
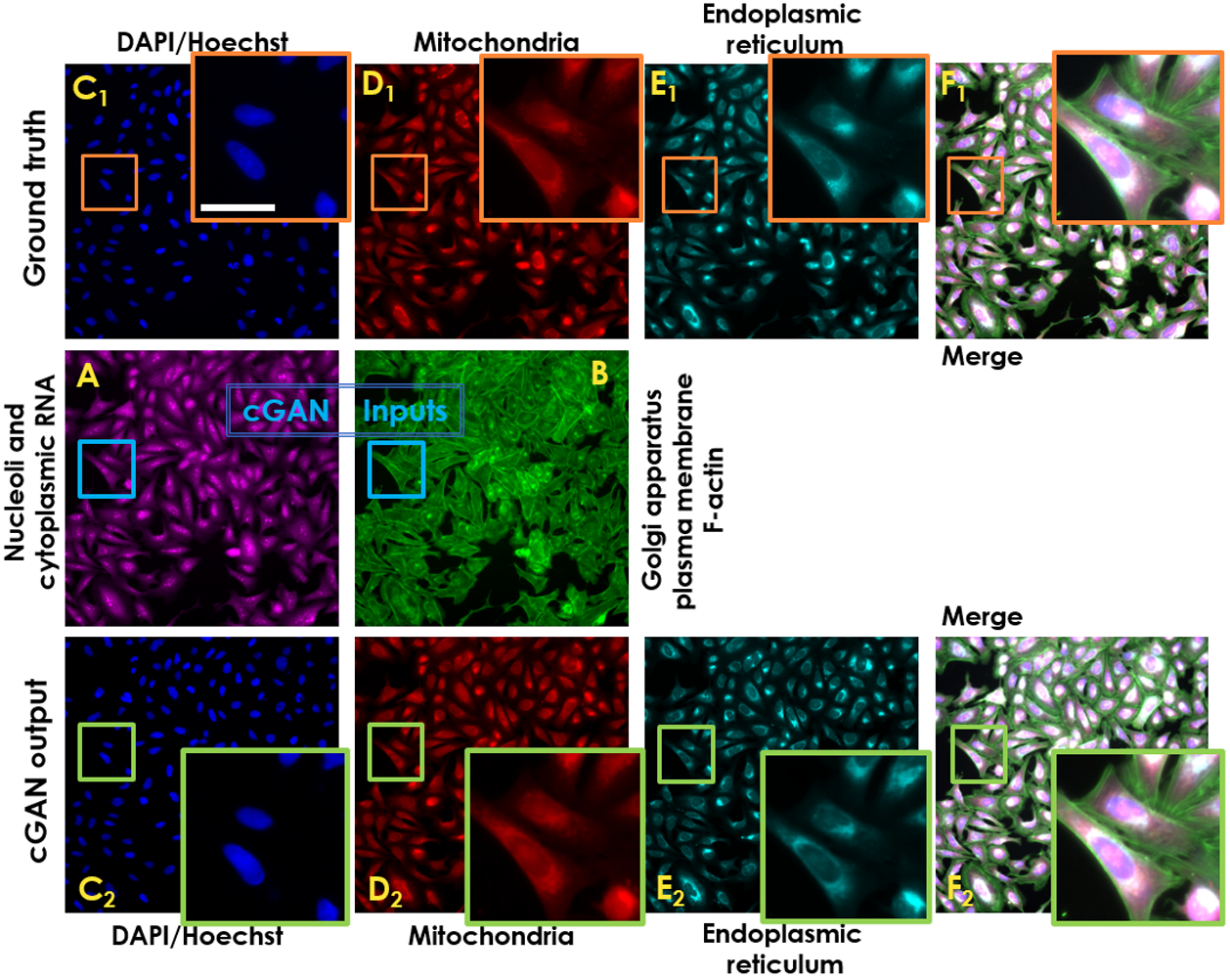
Deep-learning-based fluorescence signal prediction of U2OS-CPS cells on testing data: (a), (b) nucleoli + cytoplasmic RNA and Golgi apparatus + F-actin are used as the input of the cGAN model. ($c_{1}$), ($d_{1}$), and ($e_{1}$) The targeted fluorescent images corresponding to [DAPI/Hoechst, mitochondria, and endoplasmic reticulum, respectively, as ground truth and their cGAN corresponding prediction ($c_{2}$), ($d_{2}$), and ($e_{2}$), respectively]. ($f_{1}$), ($f_{2}$) Merged-channel images [from (a), (b), ($c_{1}$), ($d_{1}$), ($e_{1}$)] and [(a), (b), ($c_{2}$), ($d_{2}$), ($e_{2}$)] for ground truth and prediction, respectively. Scale bar is 25  μm.

In previous studies,^16^^,^^17^ predicting fluorescent proteins from transmitted microscopy signals was carried out successfully. Based on our models’ prediction performance, we propose that this occurs when the predicted protein localization is highly correlated to well-defined scattering interfaces. These findings strengthen DCNN as a state-of-art-the method in image transformation but also set limitations for protein signal prediction from nonlabeled image modalities. In the proposed work, we predicted individual fluorescence channels by inputting two-channel fluorescent labels into the DCNN models, e.g., Golgi apparatus (channel 1), membrane + nucleoli/cytoplasmic RNA (channel 2) to predict mitochondria, nucleus, and endoplasmic reticulum (see Fig. 7). All organelles were acquired from a microscopy assay imaging system using Cell Painting staining protocol,^20^ with six stains imaged across five channels, revealing eight cellular components/structures. Due to the huge amount of data collected, some of the images are out-of-focus and cannot be readily used in any data-driven analysis, especially in Golgi apparatus plasma and membrane F-actin. Hence, a necessary AF DCNN model (described in Sec. 3.1) was developed to predict focused images to be used for training the Fluo-Fluo 2, 3, 4 models to perform any data-driven analysis.

**Fig. 8:**
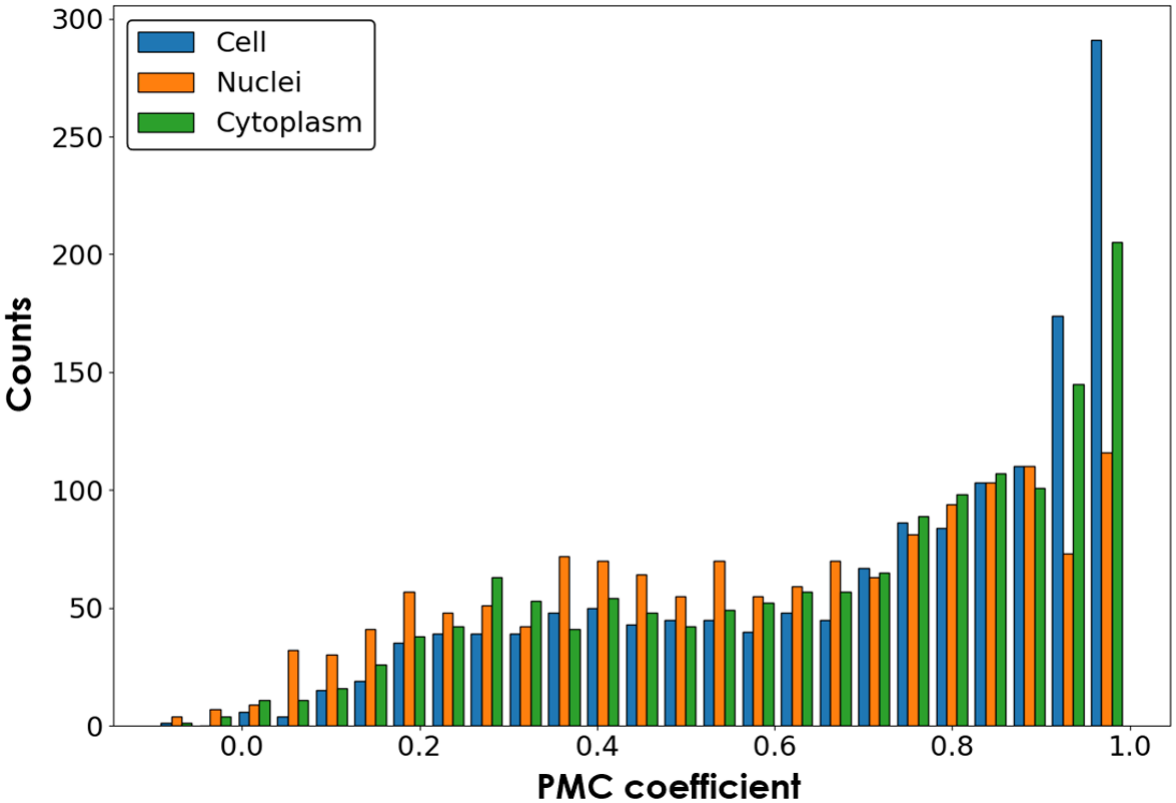
Histogram of PMC across 96 images of each feature measurement group (correlation, granularity, intensity, neighbors, radial distribution, and texture) distributed across three compartments: cell, nuclei, and cytoplasm.^29^^,^^30^

To measure the performance of the proposed models, we computed MAE, PSNR, and SSIM on 96 predicted mitochondria, nucleus (DAPI/Hoechst), and endoplasmic reticulum images and their corresponding ground truth fluorescence images in the testing dataset of U2OS-CPS (Fig. S3 in the Supplementary Material). The average scores are: MAE [0.0023, 0.0106, 0.0068], PSNR [48.1931, 37.7700, 41.3456], and SSIM [0.9772, 0.9564, 0.9731], for DAPI/Hoechst, Endoplasmic reticulum, and Mitochondria label prediction, respectively. These results are comparable to those found in Ref. 24. Following the feature measurement method provided previously^19^^,^^23^ that calculated the biological relations into feature scores among five fluorescence channels, we repeated this method with a modified pipeline for a set of five-channel images (96 images from U2OS-CPS testing dataset) and calculated the feature scores for each sample (one five-channel image) as reference. On the other hand, from the same testing dataset, the extracted two-channel images (as inputs) and their predicted three-channel images (as outputs) from the Fluo-Fluo 2, 3, 4 models form new five-channel images. Feature scores from these new images were calculated with the same pipeline as mentioned above and were compared with reference scores using the Pearson product-moment correlation coefficient (PMC) (Fig. S4 in the Supplementary Material). Each PMC was calculated across 96 samples for a single feature measurement. One sample is a region of cell to be taken from five fluorescent channels. Figure 8 and Fig. S5 in the Supplementary Material show the PMC coefficient of each feature measurement, distributed in cell, nuclei, and cytoplasm property groups, across 96 images from the U2OS-CPS testing dataset between the original five channels and hybrid-virtual two + three channels and their histograms, respectively. Ignoring the prefect correlation of 1 (self-correlation from inputs [Golgi apparatus (plasma), membrane (F-actin) + nucleoli/cytoplasmic RNA] only), the histograms show high correlation in feature measurement between the original channels and the hybrid-virtual channels, which demonstrates the reliability of using virtual channels for biological analysis. Extracted feature measurements including correlation, granularity, intensity, radius distribution, size and shape, and texture are shown in Method Section (Sec. 2) and Figs. S5 and S6 in the Supplementary Material.

The majority of correlations between features from the original channels and three prediction channels (cell, nuclei, and cytoplasm compartments) plus two original channels as inputs were strong, as seen in both the Pearson’s product-moment correlation coefficient matrices (Fig. S5 in the Supplementary Material) and its histogram (Fig. 8). Both show a significant number of features with high correlation, indicating good prediction. This also suggests that fluorescence data from multiple channels, instead of just a single channel, provide additional performance to fluorescence signal prediction tasks using DL algorithms. Further, fluorescence labeling experiments with five labels are difficult to achieve due to channel crosstalk, nonspecific binding of labels/background, and other artifacts during specimen preparation, processing, and image acquisition. Use of two to three labels simultaneously is more feasible. Prediction of the remaining three channels in a Cell Painting-type of multilabel experiment^20^ is one potential use case of cGAN image regression that may be more feasible than one-input-to-one-output channel prediction.

## 4 Discussions

Three potential applications of the algorithms developed in this work are (1) deblurring of fluorescence images, (2) prediction of fluorescence signals related to an input fluorescence signal, and (3) transfer learning in which predicted and ground truth signal differences are attributable to an altered condition of the cells in the test dataset versus the training dataset. Unlike imaging techniques capable of focusing through a large depth-of-field such as holography, fluorescence microscopy lacks the capability for image propagation, which is necessary for digitally obtaining images at different axial planes. Traditional image focusing techniques such as deconvolution methods can be employed for fluorescence microscopy with varied success.^31^ Other related methods such as multifocal microscopy could also be used to acquire the focal plane image.^32^^,^^33^ These techniques are complex themselves and/or require special instrumentation. Wu et al.^34^ showed that DCNN can be used to propagate images from a single plane to other planes that results in the possibility of acquiring a virtually focused 3D volume. Previous studies have recently developed DCNN-based methods for autofocusing resulting in quantitative out-of-focus levels.^35^^–^^37^ In previous work, our proof-of-concept used image regression-based autofocusing.^38^ Recently, Guo, et al.^39^ accelerated the iterative deconvolution process for defocusing biomedical images via deep learning. In the present work, we fully developed a DCNN-based method that can inherently learn the optical properties governing intensity-based fluorescence signal wave propagation for a large out-of-focus range of [−10  μm,10  μm] to obtain a virtual fluorescence image at the focus plane. With the advantage of not using mechanically translating hardware or extra refocusing algorithms, this proposed end-to-end technique can improve the robustness of automated microscopy and imaging systems such as integrated microplate microscopy or digital slide scanning to acquire large-scale data. This also avoids phototoxicity and photobleaching from extended imaging during manual focus adjustments that are a major concern during fluorescence microscopy experiment imaging cells. The AF algorithm (Figs. 3 and 4) is currently applicable, for example, to large digitally scanned fluorescence images in which some parts are in focus (serving as the training dataset) and other portions are out-of-focus due to errors in optics, registration, specimen alignment on the stage, or specimen mounting. Accurate prediction of fluorescence signals with cGAN remains difficult, but this work points toward conditions for better prediction performance: use of multiple fluorescence channels as training data and tight interactions between labeled molecules/compartments in the input and prediction channels (such as F-actin and vinculin). Furthermore, the tolerance level [Eq. (7), Fig. 6, and Fig. S2 in the Supplementary Material] tracks serious errors, such as spurious objects in prediction images, balanced by less important errors, such as differences in background levels. An example of transfer learning as a future application of the algorithms of this study would be to predict vinculin from F-actin signals using cells attached to glass as a training dataset and then applying the trained algorithm to cells attached to a different substrate, such as a natural biomaterial. The differences between prediction and ground truth in this case may be instructive.

Using the trained AF model, we have predicted the focused images of the testing U2OS-AF dataset, which only contains F-actin. To check the generalizability of the trained model, we applied it directly on a completely new dataset combining blurred signal from labels of Golgi apparatus + F-actin in the U2OS-CPS dataset without using transfer learning (Fig. 4 shows a typical result). In fact, both types of images have some similar characteristics, so the model can detect a similar set of features when using the same kernel filter. However, the proposed AF model performs less successfully on other channels in the U2OS-CPS dataset. AF other label channels in the U2OS-CPS dataset using the U2OS-AF algorithm pretrained on F-actin likely gives less accurate results because other channels contain different spatial features than F-actin labels that reduce the prediction accuracy of the model. Thus transfer learning should be used if the ground truth of these corresponding channels exists. If not, unsupervised domain adaptation^40^ would be a potential solution. In this study, we only performed autofocusing as a preprocessing step for images with blurred Golgi apparatus + F-actin signals in the U2OS-CPS dataset before training the Fluo-Fluo 2, 3, 4 models.

Recent research efforts have been developed to predict fluorescence images from unlabeled images using deep neural network, such as from bright field or phase contrast images.^16^^,^^17^ In our work, we sought to determine whether a network could generate a more complicated labeling of, for example, F-actin and vinculin from phase contrast images, as shown in Fig. 5. Phase contrast is a common bright-field microscopy technique used to detect details of semitransparent living cells having a wide variation of refractive index due to subcellular organelles. For example, phase contrast image features from the nucleus region are high contrast due to sharp interfaces and density fluctuations, attributable to the nuclear envelope and objects in the nucleus itself (such as nucleoli) as well as perinuclear objects in the cytoplasm. This information was useful for predicting fluorescence labels of DNA, which was largely restricted to the nucleus. In general, phase contrast microscopy using a high numerical aperture objective will provide great contrast and detail of membrane-bound organelles and is expected to accurately predict fluorescence labels of such organelles.^41^^,^^42^ On the other hand, nanoscale structures such as cytoskeletal proteins (e.g., F-actin) and adhesion proteins (e.g., vinculin) share similar contrast as the cytoplasm background, making them poor predicted signals from phase contrast microscopy inputs to the cGAN model. However, cytoskeleton structures like F-actin are more effective inputs to the cGAN model to predict the vinculin signal, likely because F-actin and vinculin share spatial connectedness in the cell, related to their coordinated mechanobiological function in linking focal adhesions to contractile apparatus of the cell.^43^ Vinculins are membrane-adjacent cytoskeleton proteins that cap and bind actin filaments to either provide or prevent connections to other F-actin binding proteins, promoting cell–cell or cell–extracellular matrix contacts.^44^ Furthermore, a computational model based on vinculin–actin binding lifetime in lamellipodia was recently proposed. The bonds between F-actin and vinculin can be formed directionally and asymmetrically, suggesting high correlation in terms of the two proteins’ spatial distributions.^43^ Hence, we suggest that the high performance of the Fluo-Fluo 2, 3, 4 models with specified inputs to predict the fluorescence label channels in the U2OS-CPS dataset are based on high spatial correlation between the underlying labeled molecules’ distributions (see Table 1).

**Table 1:** Summary of implemented models’ performances. Models (*) were implemented for feature prestudying.

Models	Trained on [inputs]–[ground truth]	Fluorescence channel predictions								
		MDA-MB-231			U2OS-AF	U2OS-CPS				
		D/H	Vin	Fa	Fa	D/H	Mito	ER	NCR	GP-Fa
PhC-Fluo 1	[PhC]–[D/H(231)]	H								
PhC-Fluo (*)	[PhC]–[Fa]			L						
PhC-Fluo 2	[PhC]–[Vin]		L-A							
Flou-Fluo 1	[Fa]–[Vin]		A-H							
AF	[Fa]–[Fa(AF) or all channels CPS]				H	L-A	L-A	L-A	L-A	H
Flou-Fluo 2	[Gp-Fa+NCR]–[DP/H(CPS)]						H			
Flou-Fluo 3	[Gp-Fa+NCR]–[Mito]							H		
Flou-Fluo 4	[Gp-Fa+NCR]–[ER]								H	
Flou-Fluo (*)	[Mito]–[D/H(CPS) or ER or GP-Fa]						A-H		A	L
Flou-Fluo (*)	[GP-Fa]–[D/H(CPS)]						L-A			
Flou-Fluo (*)	[NCR]–[D/H(CPS)] or [Mito]						A	A		

H, high; A, average; L, low performance; PhC, phase constrast; D/H, DAPI/Hoechst; Vin, vinculin; Fa, F-actin; Mito, mitochondria; ER, endoplasmic reticulum; NCR, nucleoli and cytoplasmic RNA; and GP-Fa, Golgi apparatus plasma membrane and F-actin.

The index of normalized, summed predicted image spatial and intensity error (the tolerance level) is an image-centric and interpretation-focused way to standardize comparisons of algorithm performance for deep learning image regression across multiple developers. For example, two other recent works predict fluorescence image outputs from ground truth brightfield images serving as input to deep learning algorithms.^16^^,^^17^ The image-wise Pearson correlation coefficient, while simple, does not highlight specific subregions in the image where the algorithm performs well or poorly. Further, difference images (predicted minus ground truth) are qualitative and difficult to assess, even with well-chosen colormaps. The index proposed in this study measures two competing errors with intuitive visual interpretations: pixel intensity mismatch and area mismatch. As the tolerance for intensity mismatch normalized to the mean signal intensity of ground truth becomes larger, the area mismatch normalized to the signal image area fraction tends to become smaller. Not only would this sum of normalized errors standardize reporting across laboratories and algorithm developers, the index is also adaptable to specific uses by choosing the relative weights of intensity and spatial error. This error metric could be used as a target to minimize during training the algorithm on new data.

## 5 Conclusions

The presented methodology of image regression has a great potential to reduce time and cost of microscopy studies of cells. One of the advantages of using computational microscopy through DCNNs is to allow for a single well-tuned training process to transform fluorescence images of a certain fluorescence channel into their other fluorescence images. Training the dataset is a required step, but it is performed only once with less preprocessing. Recent related research has demonstrated that overall cell morphology and that of many organelles can be predicted from transmitted light imaging. In this work, we have extended the use of deep neural networks to predict subcellular localization of proteins from other proteins from co-registered images. Finally, the proposed DCNN will be a cost-effective tool for many biological studies involving protein–protein and organelle–protein relationships and will help researchers visualize the coordination of subcellular features under a variety of conditions aiding in understanding of fundamental cell behaviors.

In future work, the algorithms developed here could be used to perform real-time virtual organelle self-coding for live-cell and intravital video microscopy governed by an inferencing device integrated to the microscope.
