# Supplementary material for: Virtual organelle self-coding for fluorescence imaging via adversarial learning
Source: J Biomed Opt. 2020 Sep 29;25(9):096009. doi: 10.1117/1.JBO.25.9.096009 (PMC7522603; doi:10.1117/1.JBO.25.9.096009)
Supplement: Supplementary file 1 [file JBO_025_096009_SD001.pdf]

Supplementary Information

Virtual organelle self-coding for fluorescence imaging via adversarial learning

Thanh Nguyen[1,*], Vy Bui[1], Anh Thai[1], Van Lam[2], Christopher Raub[2], Lin-Ching Chang[1], George Nehmetallah[1]

[1] Electrical Engineering and Computer Science Department, The Catholic University of America, Washington DC, 20064, USA
[2] Biomedical Engineering Department, The Catholic University of America, Washington DC, 20064, USA

*Correspondence: 32nguyen@cua.edu

Methods

Computational pipeline with CellProfiler:

Original pipeline could be found at [20], modified version that we used can be found at: https://github.com/32nguyen/SupplementaryMethod_CellProfiler

Python codes to extract the feature data automatically if not familiar with MySQL. Example of feature measurement is also provided to observation. Original dataset could be downloaded at https://data.broadinstitute.org/bbbc/BBBC025/,

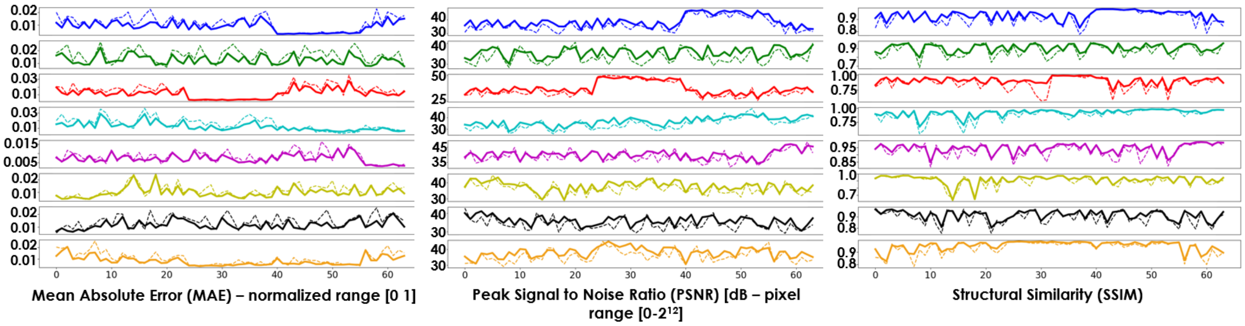

Supplemental Fig. 1: MAE, PSNR, and SSIM on 64 predicted images vs. focused ground truth images per z depth (-10, -8, -6, -4, 4, 6, 8 and 10µm, top to bottom) with AF model's prediction , dash line is out-focus image v/s ground truth, solid line is predicted image v/s ground truth.

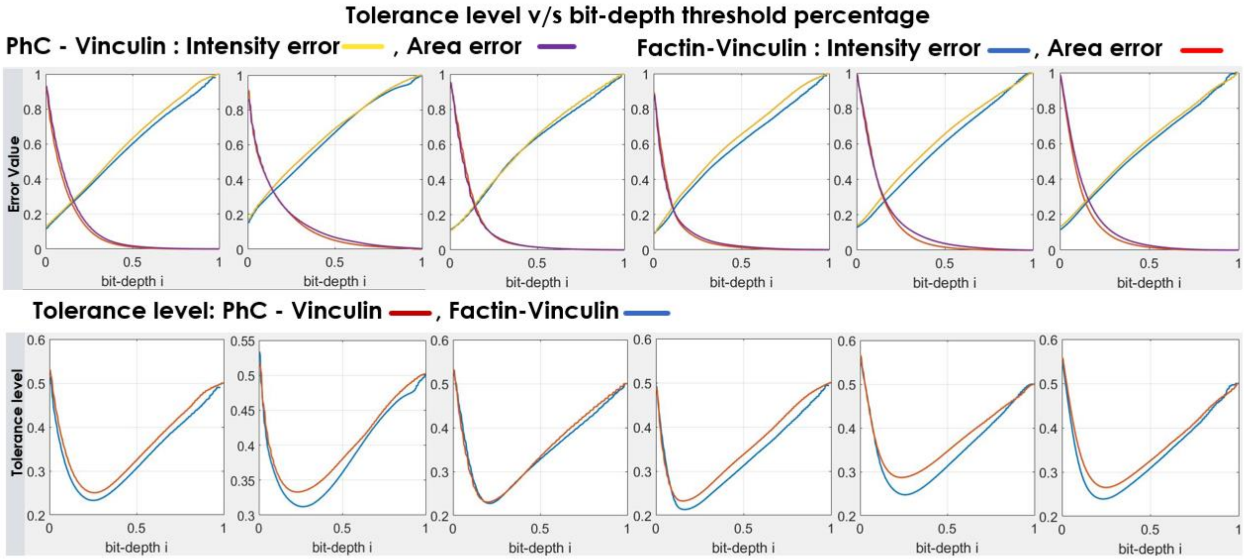

Supplemental Fig. 2: Tolerance level (y-axes) v/s bit-depth threshold percentage (x-axes) computed on 6 testing images (left to right). The first row is the intensity error (*IE*) versus area fraction of intensity-based segmentation error (*SE*) of both PhC-Fluo 2 and Fluo-Fluo 1 models. The second row is the summation of equally-weighted *IE* and *SE* (β_1=β_2=0.5) of both PhC-Fluo 2 and Fluo-Fluo 1 models.

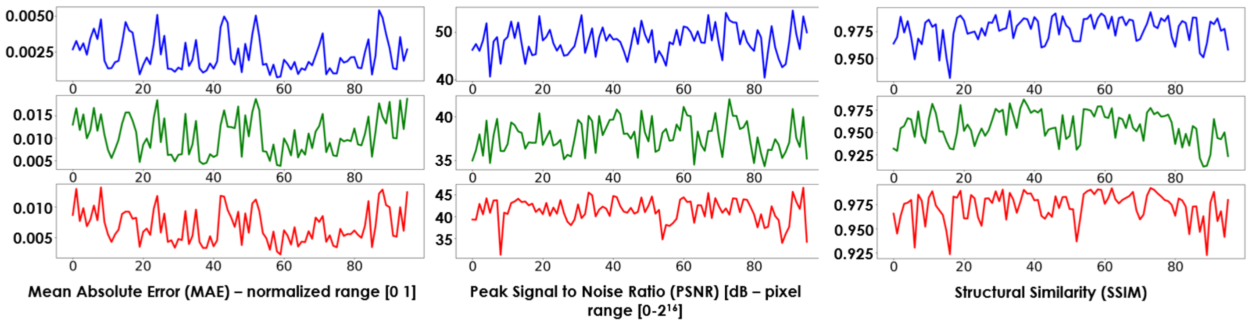

Supplemental Fig. 3: MAE, PSNR, SSIM on 96 predicted nucleus DAPI/Hoechst, Endoplasmic reticulum and Mitochondria images vs. corresponding ground truth fluorescence images in testing dataset of U2OS-PS. Top to bottom are for DAPI/Hoechst, Endoplasmic reticulum, and Mitochondria, respectively.

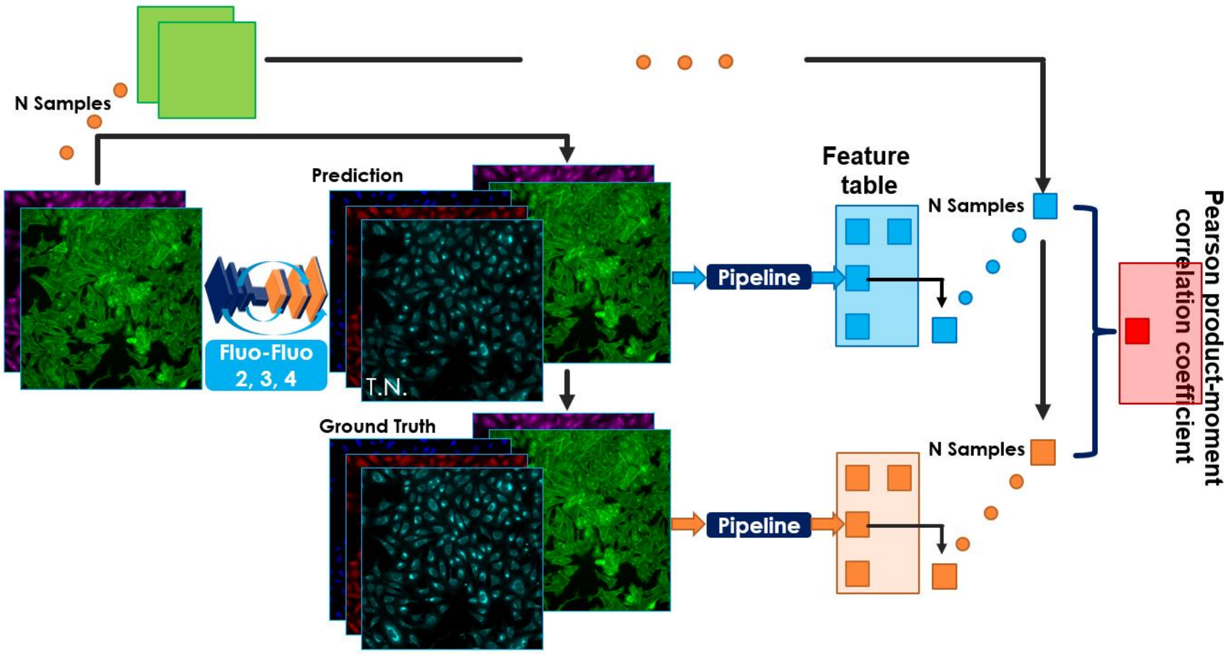

Supplemental Fig. 4: Procedure for Pearson product-moment correlation coefficient extracted from feature measurement table. Each feature type in table from original (5) channels and hybrid-virtual (2+3) channels crossing N samples (each sample is an image which has 5 channels) to compute the Pearson product-moment correlation coefficient.

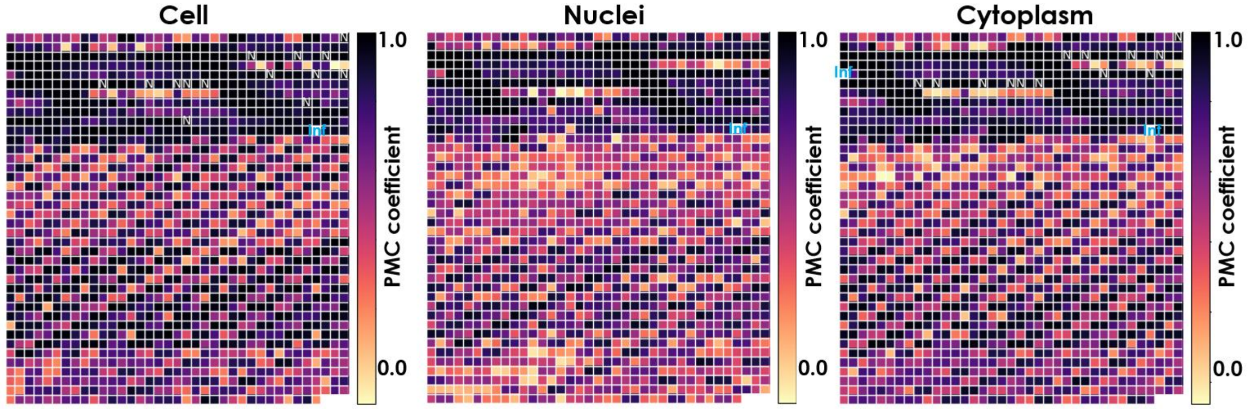

Supplemental Fig. 5: Pearson product-moment correlation coefficient of each feature measurement across 96 images on testing data between original (5) channels and hybrid-virtual (2+3) channels. "N" marks the correlation coefficients of features measured only on 2-channel input images in both cases (Golgi apparatus + F-actin) which results perfect correlations. "Inf" marks un-resolved correlation due to 0-division in measurements. Those features are distributed across 3 compartments: Cell, nuclei, and cytoplasm [20] (see Supplement - Methods for feature's organization and Supplement Fig. 6 for full feature measurements).

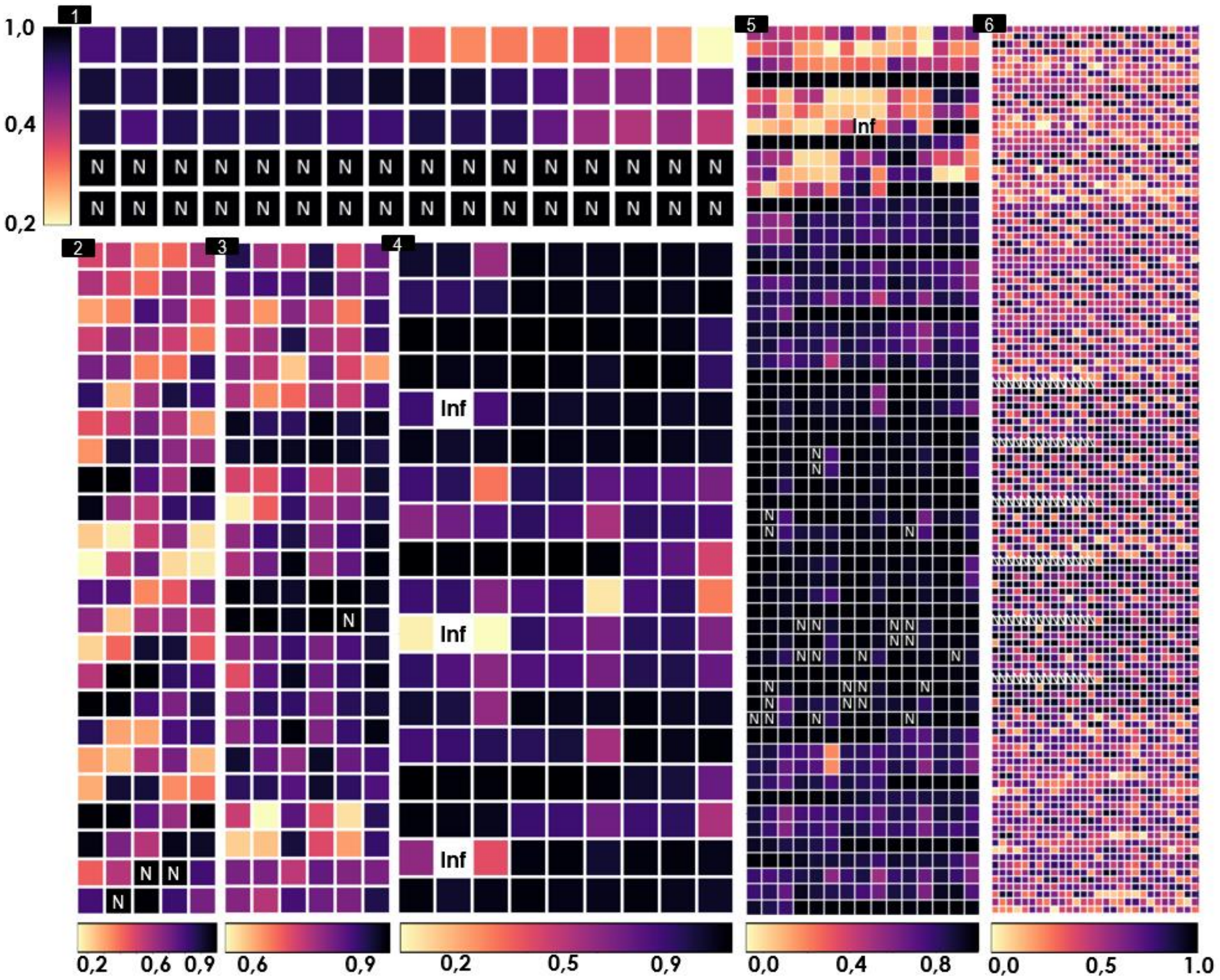

Supplemental Fig. 6: Pearson product-moment correlation coefficient of each feature measurement across 96 samples on testing data between original 5 channels and 2+3 channels (2 of inputs and 3 of predictions). "N" marks the correlation coefficients of features measured only on 2-channel input images in both cases (Golgi apparatus + F-actin) which results perfect correlations. "Inf" marks un-resolved correlation due to 0-division in measurements. Following #1-6, PMC coefficient is following 6 feature groups: Granularity, correlation, radial distribution, size-shape, intensity, and texture. Correlation coefficient grids were re-organized as the last axis index changing fastest.
